# Clinical and pathological outcomes of induction chemotherapy before neoadjuvant radiotherapy in locally‐advanced rectal cancer

**DOI:** 10.1002/jso.25474

**Published:** 2019-04-16

**Authors:** Karin M. Hardiman, Alexis G. Antunez, Arielle Kanters, Ari D. Schuman, Scott E. Regenbogen

**Affiliations:** ^1^ Department of Surgery, Division of Colorectal Surgery Michigan Medicine Ann Arbor Michigan; ^2^ University of Michigan Medical School Ann Arbor Michigan; ^3^ Department of Surgery Michigan Medicine Ann Arbor Michigan; ^4^ Center for Healthcare Outcomes and Policy Ann Arbor Michigan

**Keywords:** induction chemotherapy, rectal neoplasm, survival

## Abstract

**Background and Objectives:**

In North America, preoperative combination chemoradiation is the most commonly recommended and utilized approach to locally advanced rectal cancer. There is increasing interest in the use of induction chemotherapy (IC) before radiation and surgery in locally advanced rectal cancer. How widely IC is being used and whether it improves pathologic and oncologic outcomes is unknown.

**Methods:**

We evaluated clinical stage 2 or 3 rectal cancer patients in the National Cancer Database between 2006 and 2015. We identified predictors of use of IC with multivariable logistic regression and compared survival between groups using Cox proportional hazards regression.

**Results:**

Among 36 268 patients, IC use increased significantly over time from 5.5% in 2006 to 15.9% in 2015 (*P* < 0.001). Treatment at a hospital with a high IC rate was an independent predictor of receipt of IC. IC and traditional therapy yielded similar pathologic complete response rates (32.2% vs 30.5%, *P* = 0.2) and similar 5‐year survival (82.4% vs 81.4%, 0.71).

**Conclusions:**

Use of IC for locally advanced rectal cancer has increased significantly. The choice of IC seems to be driven more by institutional and regional practice patterns than clinical characteristics and is not associated with improved pathologic or oncologic outcomes.

**Abbreviations**
5‐FU: 5‐fluorouracil
FOLFOX: 5‐FU with leucovorin and oxaliplatin
IC: induction chemotherapy

## 1 INTRODUCTION

Optimal management of locally advanced rectal involves multimodality therapy including chemotherapy, radiation, and surgery. Since the publication of the German Rectal Cancer Trial,^1^, ^2^ the most commonly recommended practices have changed from postoperative combination chemoradiation to preoperative combination chemoradiation and adjuvant systemic chemotherapy. Adherence to neoadjuvant chemoradiotherapy remains inadequate; however, with significant variation in treatment based on center type, volume, and geographic location.^3^ More recently, single‐center studies have reported the use of induction chemotherapy (IC) before combination chemotherapy and radiation followed by surgery,^4^, ^5^ with the goals of introducing systemic therapy earlier in the course of treatment, and potentially increasing the rate of complete pathologic response.^6^ Others have endorsed the delivery of all chemotherapy and radiation before surgery, recognizing that surgical complications preclude adjuvant chemotherapy in up to 34% of patients.^7^, ^8^ There is only a small phase 2 randomized trial comparing IC to adjuvant chemotherapy, which did not identify a difference in pathologic complete response or survival between groups.^9^ Despite this, the National Comprehensive Cancer Network Guidelines now include IC among endorsed options for treatment of stages 2 and 3 rectal cancer.^10^

To date, large scale or randomized studies comparing IC against standard preoperative chemoradiation are lacking. The National Cancer Institute‐supported PROSPECT trial randomized patients to standard chemoradiation or IC with the selective omission of preoperative radiation, but outcomes of this trial are still forthcoming.^11^ On the one hand, IC might induce the more preoperative response, reducing the likelihood of local failure, and treating occult metastatic disease earlier. On the other hand, delayed surgery might allow local expansion and worsen the likelihood of surgical margin clearance and leave more time for the primary tumor to metastasize. The real‐world outcomes of the IC strategy cannot be assessed without population‐based evaluation outside of highly‐selected case series. The prevalence of IC use outside of the highly specialized institutions that have reported its use is unknown.

In this study, we used the National Cancer Database (NCDB), which includes data from all American College of Surgeons Commission on Cancer accredited hospitals, accounting for approximately 70% of all cancer patients in treated US hospitals.^12^ This population‐based cohort offers a realistic epidemiologic assessment of the outcomes of patients with locally advanced rectal cancer nationwide. We used data on timing of chemotherapy, radiation and surgery to classify therapy as either IC before radiation or traditional concurrent chemoradiation before surgery. We sought to understand time trends and patient and provider characteristics associated with the use of IC, and the clinical and pathologic outcomes of IC compared with traditional chemoradiation.

## 2 MATERIALS AND METHODS

### 2.1 Patient selection

We queried the NCDB Participant Use File from 2006 to 2015 and identified all patients with clinical stage 2 or 3 rectal cancer. The analysis was limited to patients who underwent preoperative chemoradiation and surgery for invasive adenocarcinoma, mucinous adenocarcinoma, or signet ring cell carcinoma of the rectum with curative intent.

**Figure 1:**
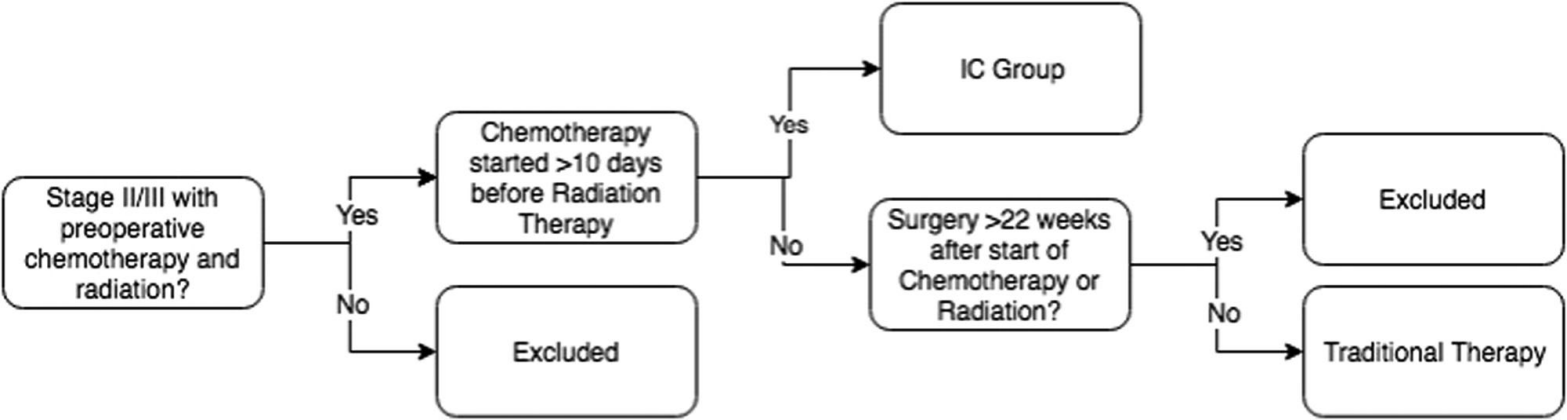
Definitions of induction chemotherapy (IC) and traditional therapy groups of stages 2 and 3 rectal cancer patients

Among patients who received radiation and/or chemotherapy as initial course of therapy, we defined two groups of interest using variables available in the NCDB (1) the IC group was defined as patients who received chemotherapy separate from radiation before surgery and (2) traditional therapy was defined as patients who received concurrent chemotherapy and radiation before surgery (Figure 1). The NCDB records patients as having received chemotherapy if they receive any type at any time, and characterizes chemotherapy as either preoperative or postoperative. It does not distinguish chemotherapy regimens and thus cannot differentiate 5‐FU with leucovorin and oxaliplatin (FOLFOX), for example, from single agent 5‐fluorouracil (5‐FU) or capecitabine. Thus, to identify patients who received IC separately from radiation, we used the timing of initiation of chemotherapy compared with the timing of initiation of radiation therapy. Examination of the interval between initiation of chemotherapy and radiation revealed a clear transition point at the 10‐day mark, whereby patients who appeared to have received combined chemoradiation were generally clustered around a fewer than 10‐day difference, distinct from a group that had start times much greater than 10 days. IC patients were thus defined as stage 2 or 3 patients who received both preoperative chemotherapy and radiation, but with start dates greater than 10 days apart. The traditional therapy patients were defined as those who started chemotherapy and radiation concurrently less than 10 days apart—in fact, a majority of these patients started both on the same day. To further specify the comparison group of traditional chemoradiotherapy followed by surgery, we excluded patients who underwent surgery greater than 22 weeks after chemotherapy and radiation, as these likely represent patients who initially refused surgery, had substantial complications of therapy, or initially pursued nonoperative management.

### 2.2 Statistical analysis

Descriptive statistics were used to compare characteristics of patients between treatment groups, using *χ* ^2^ tests for categorical variables, the Student *t* test for continuous variables, and analysis of variance for multicategory comparisons of continuous data. We identified independent predictors of receiving IC using multivariable logistic regression, including patient factors (age, race, rectal cancer stage, place of residence, income, type of insurance, and receipt of postoperative chemotherapy) and hospital factors (regional location and facility type) that were significant in the univariate analysis. We categorized the hospital rate of IC into quartiles and included this in the model to account for the role of institutional practice patterns. We compared overall survival between therapy regimens using multivariable Cox proportional hazard regression, adjusting for the same patient and hospital factors as above, and applying robust standard errors to account for clustering of outcomes within hospitals. All statistical analyses were conducted using STATA version 14 (StataCorp LP, College Station, TX).

## 3 RESULTS

### 3.1 Patient and hospital characteristics

**Figure 2:**
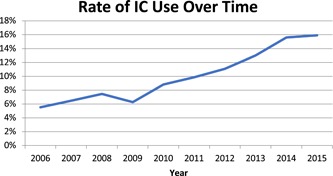
The percentage of patients receiving induction chemotherapy (IC) increased over time [Color figure can be viewed at wileyonlinelibrary.com]

Of 36 268 patients included in the analysis, 3241 (8.9%) received IC. The proportion of patients receiving IC increased significantly over time (Figure 2), from 5.5% in 2006 to 15.9% in 2015 (*P* < 0.001). The annual rate of increase was greatest between 2011 and 2014.

### 3.2 Predictors of receipt of IC

Comparisons of patient characteristics in IC and traditional therapy groups are shown in Table 1. Patients who received IC were slightly younger (58.6 vs 59.7, *P* < 0.001), and more likely to be non‐white (24.8% vs 18.5%, *P* < 0.001), have Medicaid (8.1% vs 6.8%, *P* < 0.001), residing in a zip code in the top or bottom income bracket (53.4% vs 47.4%, *P* < 0.001) and lowest education bracket (19.3% vs 15.9%, *P* < 0.001), and residing in a metropolitan area (83.3% vs 78.1%, *P* < 0.001). IC was also more common among those with clinical stage 3 disease (59.6% vs 53.6%, *P* < 0.001).

**Table 1:** Patient characteristics of stage 2 and 3 rectal cancer who received traditional therapy or induction chemotherapy (IC)

Patient characteristics	Traditional (N = 33 012)	IC (N = 3241)	*P* value
Mean age (SD), n (%)	59.7 (12.2)	58.6 (12.3)	<0.001
<50 years old	7676 (23.3)	836 (25.8)	
50‐60 years old	9694 (29.4)	978 (30.2)	
60‐70 years old	8949 (27.1)	863 (26.6)	
70 + years old	6693 (20.3)	564 (17.4)	
Female	12 386 (37.5)	1216 (37.5)	1.0
Race/ethnicity, n (%)			<0.001
White	28 754 (87.1)	2717 (83.8)	
Black	2487 (7.5)	324 (10.0)	
Asian	1323 (4.0)	143 (4.4)	
Other	448 (1.4)	57 (1.8)	
Hispanic	1777 (5.6)	269 (8.6)	
Insurance, n (%)			<0.001
Private insurance	17 113 (51.8)	1713 (52.9)	
Medicare	11 248 (34.1)	991 (30.6)	
Medicaid	2248 (6.8)	263 (8.1)	
Other government insurance	488 (1.5)	43 (1.3)	
Not insured	1509 (4.6)	189 (5.8)	
Income quartiles, n (%)^a^			<0.001
<38K	5593(17.0)	595 (18.5)	
38K‐48K	8244 (25.1)	680 (21.2)	
48K‐63K	9017 (27.5)	819 (25.5)	
>63K	9963 (30.4)	1120 (34.9)	
Education, n (%)^a^ ^,^ ^b^			<0.001
≥29% (lowest edu)	5098 (15.9)	602 (19.3)	
20%‐28.9%	7.738 (24.2)	733 (23.4)	
14%‐19.9%	7979 (25.0)	714 (22.8)	
<14% (highest edu)	11 165 (34.7)	1079 (34.5)	
Urban/rural patient location, n (%)			<0.001
Metropolitan	25 173 (78.1)	2595 (83.3)	
Urban/Suburban	6166 (19.1)	443 (14.2)	
Rural	911 (2.8)	76 (2.4)	
Average distance travelled to hospital (SD), miles	31.5 (104.7)	30.3 (102.4)	0.56
Rectal cancer clinical stage			<0.001
Stage 2	15 305 (46.4%)	1311 (40.5%)	<0.0001
T3	13 342	1076	
T4a	559	64	
T4b	453	78	
Stage 3	17 707 (53.6%)	1930 (59.6%)	<0.001
T1/2N1	2791	146	
T1N2a	19	4	
T1/2N2b	20	5	
T2/3N2a	1966	226	
T3/4aN1	10 499	1299	
T4aN2a	178	26	
T3/4aN2b	277	43	
T4bN1/2	603	105	
Charlson/Deyo score, n (%)			0.35
0	26 314 (79.7)	2588 (79.9)	
1	5383 (16.3)	516 (15.9)	
2	1001 (3.0)	96 (3.0)	
3	314 (1.0)	41 (1.3)	
Stage 4 on pathology	434 (2.6)	52 (3.1)	0.18
Receipt of postoperative chemotherapy	11 513 (34.9)	941 (29.0)	<0.001
Path stage 2/3	6415	512	
Path stage 4	167	18	
Complete regression on pathology, n (%)	3698 (30.4)	370 (32.2)	0.2

^a^ Assigned by zipcode of patient's residence.

^b^ Proportion of population without high school degree.

Univariable comparisons of hospital characteristics treating rectal cancer patients in the IC or traditional therapy groups is shown in Table 2. Patients receiving IC were more likely to be treated in an Academic/Research Program Hospital (42.4% vs 32.8%, *P* < 0.001) and more likely to be treated in the Middle Atlantic region (18.6% vs 12.7%, *P* < 0.001).

**Table 2:** Characteristics of hospitals treating stage 2 and 3 rectal cancer who received traditional therapy or induction chemotherapy (IC)

Hospital characteristics	Patients receiving treatment in hospital type/location		
Hospital type	Traditional, n (%)	IC, n (%)	<0.001
Community Cancer Program	2831 (9.0)	216 (7.1)	
Comprehensive Community Cancer Program	13 985 (44.5)	1165 (38.4)	
Academic/Research Program	10 929 (34.8)	1,287 (42.4)	
Integrate Network Cancer Program	3674 (11.7)	369 (12.2)	
Hospital region			<0.001
New England	1775 (5.7)	206 (6.8)	
Middle Atlantic	4000 (12.7)	564 (18.6)	
South Atlantic	6811 (21.7)	639 (21.0)	
East North Central	6462 (20.6)	547 (18.0)	
East South Central	1914 (6.1)	222 (7.3)	
West North Central	3342 (10.6)	181 (6.0)	
West South Central	2338 (7.4)	255 (8.4)	
Mountain	1508 (4.8)	110 (3.6)	
Pacific	3269 (10.4)	313 (10.3)	

In multivariable logistic regression analysis, displayed in Table 3, patient characteristics that were independently associated with receipt of IC included: Black (odds ratio [OR], 1.18; 95% confidence interval [CI],1.02‐1.36) or Hispanic (OR, 1.20; CI, 1.01‐1.41) race, being from a rural area (OR, 1.36; CI, 1.04‐1.79), and having stage 3 cancer (OR, 1.21; CI, 1.11‐1.31). Hospital characteristics independently associated with receipt of IC included location in the Middle Atlantic region (OR, 1.25; CI, 1.02‐1.54) and being treated in a hospital with a higher proportional use of IC (OR, 22.4; CI, 17.97‐27.92 for highest vs lowest quartile). The change in the relationship to receipt of IC between the univariate and multivariable analysis for rural patients appears to be because the vast majority of these patients (67%) receive treatment at hospitals that have low utilization of IC.

**Table 3:** Predictors of receipt of induction chemotherapy (IC) utilizing multivariable logistic regression

Patient characteristics	OR	SE			95% CI
Mean Age (SD)					
<50 years old	Reference				
50‐60 years old	1.00		0.06	0.89	1.12
60‐70 years old	1.01		0.07	0.89	1.16
70 + years old	0.90		0.07	0.76	1.05
Race/ethnicity					
White	Reference				
Black^*^	1.18		0.09	1.02	1.36
Asian	0.95		0.10	0.77	1.17
Other	1.23		0.22	0.86	1.75
Hispanic^*^	1.20		0.10	1.01	1.41
Insurance					
Uninsured	Reference				
Private Insurance	0.92		0.09	0.76	1.11
Medicare	0.95		0.11	0.75	1.20
Medicaid	0.92		0.10	0.74	1.13
Other government insurance	0.90		0.18	0.61	1.33
Insurance status unknown	0.97		0.19	0.65	1.44
Income quartiles^a^					
<38K	Reference				
38K‐48K	0.90		0.06	0.79	1.04
48K‐63K	0.98		0.07	0.84	1.14
>63K	1.12		0.10	0.94	1.33
Education^a^ ^,^ ^b^					
≥29% (lowest edu)	Reference				
20%‐28.9%	0.96		0.07	0.83	1.10
14%‐19.9%	0.97		0.08	0.83	1.13
<14% (highest edu)	0.90		0.08	0.76	1.06
Urban/rural patient location					
Metropolitan	Reference				
Urban/suburban	0.93		0.06	0.82	1.06
Rural^*^	1.36		0.19	1.04	1.79
Rectal cancer clinical stage^*^					
Stage 2	Reference				
Stage 3	1.21		0.05	1.11	1.31
*Hospital characteristics*					
Hospital type					
Community Cancer Program	Reference				
Comprehensive Community Cancer Program	0.89		0.08	0.75	1.05
Academic/Research Program	0.99		0.09	0.83	1.17
Integrate Network Cancer Program	0.94		0.09	0.77	1.15
Hospital Region					
New England	Reference				
Middle Atlantic^*^	1.25		0.13	1.02	1.54
South Atlantic	1.01		0.11	0.82	1.25
East North Central	0.98		0.11	0.80	1.21
East South Central	1.06		0.13	0.83	1.35
West North Central	1.00		0.13	0.78	1.29
West South Central	1.27		0.15	1.00	1.60
Mountain	1.33		0.19	1.00	1.77
Pacific	1.09		0.13	0.87	1.37
Hospital quartile of IC use^*^					
1st	Reference				
2nd	4.51		0.54	3.56	5.71
3rd	9.17		1.06	7.31	11.51
4th	22.40		2.52	17.97	27.92

Abbreviations: CI, confidence interval; OR, odds ratio; SE, standard error.

^a^ Assigned by zipcode of patient's residence.

^b^ Proportion of population without high school degree.

^*^ *P* < 0.05.

### 3.3 Clinical and pathologic outcomes, by treatment group

**Figure 3:**
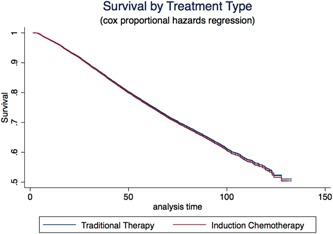
Survival analysis comparing induction chemotherapy and traditional care. Cox proportional hazard regression analysis revealed equivalent survival between patients receiving induction chemotherapy separate from radiation (IC) and those receiving traditional therapy [Color figure can be viewed at wileyonlinelibrary.com]

The proportion of patients who had complete tumor regression on pathology was not different between the IC and traditional groups (32.2% vs 30.4%, *P* = 0.20). Likewise, the unadjusted survival functions between the two treatment groups were not significantly different (*P* = 0.85). Graphical display of the Cox regression survival analysis, adjusting for patient and hospital factors, is shown in Figure 3. Adjusting for patient and hospital characteristics, the IC group had equivalent survival to the traditional care group. Five‐year survival for traditional therapy was 81.4%, while for IC, it was 82.4%, (*P* = 0.71).

## 4 DISCUSSION

In this study, we find that, despite the lack of large randomized trials to support IC, there has been a steady annual increase in its use for clinical stages 2 and 3 rectal cancer and that the primary determinant of this use is institutional practice pattern, rather than clinical indications. Patients who were Black or Hispanic, from a rural area, had stage 3 cancer, or were treated at a hospital in the Middle Atlantic region were also more likely to receive IC. We did not find; however, evidence to support an effect of IC on the likelihood of either complete pathologic response or overall survival. This study utilized data from the NCDB, which is a population‐based, nationwide sample, representing the vast majority of treated rectal cancers in US hospitals. Thus, it provides a realistic assessment of national practice patterns beyond just specialty referral centers.

The use of IC is just one of several recent changes in the prevailing treatment approaches for rectal cancer. In general, the trend has been toward more treatment being given before surgery, with increasing interest in strategies that might obviate the need for surgery altogether for some patients. Improved surgical technique, with total mesorectal excision and negative circumferential margin, along with radiation, became the standard of care after studies showed a decreased risk of local recurrence.^13^, ^14^, ^15^, ^16^ The German rectal cancer trial established that preoperative radiation was superior to postoperative treatment, less from improved survival than because of decreased toxicity.^2^ Thus, some have pursued IC to avoid failures to receive chemotherapy after surgery due to perioperative complications.^8^ In addition, Habr‐Gama showed that preoperative multimodal treatment resulted in tumor shrinkage and thus improved sphincter preservation, suggesting a role for IC in reducing the rate of permanent colostomy.^17^ In another study, some patients who achieved an apparent complete clinical response to preoperative therapy did well over time without surgery.^18^ Since that time, multiple studies aimed to improve local tumor response through increasing multimodal preoperative therapy, though randomized, multi‐institution data in support of such approaches is still lacking.^4^, ^5^, ^19^

Because there has not been a large randomized trial of the use of IC in rectal cancer, currently available studies must be interpreted with caution. In 2010 Chua et al^20^ reported on 105 patients who received 12 weeks of IC with oxaliplatin and capecitabine followed by 54 Gy of radiation over 6 weeks, then surgery, then another 12 weeks of chemotherapy and showed that this was feasible. An additional feasibility study moving all of the planned chemotherapy to before radiation was reported in 2014 from Memorial Sloan‐Kettering treating 57 patients with induction FOLFOX.^4^ This group more recently published a retrospective comparison of their single‐institution data comparing 320 patients receiving chemotherapy and radiation followed by surgery and then chemotherapy to 308 patients receiving all of their therapy before surgery.^5^ The only randomized trial was a phase 2 study from Spain which compared IC to adjuvant in a total of 108 patients and this study did not identify a difference in pathologic complete response (the primary endpoint) or survival between groups despite improved compliance with receipt of planned chemotherapy.^9^, ^21^

In summary, the use of IC may achieve one of two goals. First, sensitive tumors will shrink completely before surgery and patients will receive earlier systemic therapy. The second is that many but not all patients receive a survival benefit from FOLFOX^22^ and tumors with a greater period of time in situ will have a theoretical increased potential for metastasis before resection of the primary tumor. Two recent studies of rectal cancer patients with pathologic complete response using the NCDB found that these patients did have a survival benefit from adjuvant chemotherapy.^23^, ^24^ However, we do not yet know how to predict who these patients are and these are clearly the sensitive tumors so it follows logically that these patients would benefit. Only long‐term data from a well‐designed trial that includes detailed tumor information will help us to understand whether we are helping patients or allowing more time for metastasis by changing our treatment algorithm.

There are limitations to the current study. First, because in NCDB data, it is not possible to ascertain exact chemotherapy regimens, we do not know exactly what the patients received. Nevertheless, we applied careful, clinically reasoned assignment to treatment groups, according to criteria that distinguish patients most likely to have received systemic chemotherapy separate from radiation. Second, NCDB does not contain other endpoints such as local and distant recurrence rates which would be of interest. Third, because this was an observational study, there may be selection bias in the assignment of patients to IC vs standard chemoradiation that could affect the pathologic and oncologic outcome comparisons. However, recognizing that the most powerful predictor of use of IC was the institutional practice pattern, rather than patient characteristics, confounding by indication was likely less influential, as treatment decisions seem to have more to do with the provider than patient differences. Further, because the clinical and pathologic outcomes were nearly identical, even in this national data set, it is unlikely that clinically important confounding has altered the conclusions.

In conclusion, the use of IC for patients with locally advanced rectal cancer in NCDB is increasing over time, but it is still used in only a minority of patients in the US. Overall, the strongest predictor of treatment algorithm including IC was the treating institution's rate of use of IC, indicating that patients are receiving different treatments at different hospitals, driven primarily by local practice patterns. We found no association between the use of IC and improved overall survival or rate of pathologic complete response. Thus, prospective data are needed to better establish the role of IC in the management of locally advanced rectal cancer.

## CONFLICT OF INTERESTS

The authors declare that there are no conflict of interests.

## DATA AVAILABILITY STATEMENT

The data that support the findings of this study are available from the Commission on Cancer, the National Cancer Database (NCDB). Restrictions apply to the availability of these data, which were used under a data use agreement for this study. Data are available from the NCDB via application at: https://www.facs.org/quality‐programs/cancer/ncdb/puf.^25^

## SYNOPSIS

Induction chemotherapy (IC) separate from radiation is increasingly being used in the US despite limited evidence to support it. We queried the National Cancer Database and found that the use of IC has increased from 6% to 16% over the last 10 years for locally advanced rectal cancer, but we did not find evidence that its use improved survival or rate of complete response.

## References

[1] Sauer R , Liersch T , Merkel S , et al. Preoperative versus postoperative chemoradiotherapy for locally advanced rectal cancer: results of the German CAO/ARO/AIO‐94 randomized phase III trial after a median follow‐up of 11 years. J Clin Oncol. 2012;30:1926‐1933. 22529255 10.1200/JCO.2011.40.1836

[2] Sauer R , Becker H , Hohenberger W , Rodel C , et al. Preoperative versus postoperative chemoradiotherapy for rectal cancer. N Engl J Med. 2004;351:1731‐1740. 15496622 10.1056/NEJMoa040694

[3] Monson JR , Probst CP , Wexner SD , Remzi FH , et al. Failure of evidence‐based cancer care in the United States: the association between rectal cancer treatment, cancer center volume, and geography. Ann Surg. 2014;260:625‐631. 25203879 10.1097/SLA.0000000000000928

[4] Cercek A , Goodman KA , Hajj C , Weisberger E , et al. Neoadjuvant chemotherapy first, followed by chemoradiation and then surgery, in the management of locally advanced rectal cancer. J Natl Compr Canc Netw. 2014;12:513‐519. 24717570 10.6004/jnccn.2014.0056 PMC5612781

[5] Cercek A , Roxburgh CSD , Strombom P , Smith JJ , et al. Adoption of total neoadjuvant therapy for locally advanced rectal cancer. JAMA Oncol. 2018;4:e180071. 29566109 10.1001/jamaoncol.2018.0071 PMC5885165

[6] Smith JJ , Chow OS , Gollub MJ , Nash GM , et al. Organ preservation in rectal adenocarcinoma: a phase II randomized controlled trial evaluating 3‐year disease‐free survival in patients with locally advanced rectal cancer treated with chemoradiation plus induction or consolidation chemotherapy, and total mesorectal excision or nonoperative management. BMC Cancer. 2015;15:767. 26497495 10.1186/s12885-015-1632-z PMC4619249

[7] Cheung WY , Neville BA , Earle CC . Etiology of delays in the initiation of adjuvant chemotherapy and their impact on outcomes for Stage II and III rectal cancer. Dis Colon Rectum. 2009;52:1054‐1063. 19581846 10.1007/DCR.0b013e3181a51173

[8] Tevis SE , Kohlnhofer BM , Stringfield S , Foley EF , et al. Postoperative complications in patients with rectal cancer are associated with delays in chemotherapy that lead to worse disease‐free and overall survival. Dis Colon Rectum. 2013;56:1339‐1348. 24201387 10.1097/DCR.0b013e3182a857eb PMC3884512

[9] Fernandez‐Martos C , Garcia‐Albeniz X , Pericay C , Maurel J , et al. Chemoradiation, surgery and adjuvant chemotherapy versus induction chemotherapy followed by chemoradiation and surgery: long‐term results of the Spanish GCR‐3 phase II randomized trialdagger. Ann Oncol. 2015;26:1722‐1728. 25957330 10.1093/annonc/mdv223

[10] Network NCC. NCCN Guidelines: rectal cancer. http://www.nccn.org/professionals/physician_gls/pdf/rectal.pdf. Accessed 17 July 2018.

[11] Schrag D , Weiser MR , Goodman KA , Gonen M , et al. Neoadjuvant chemotherapy without routine use of radiation therapy for patients with locally advanced rectal cancer: a pilot trial. J Clin Oncol. 2014;32:513‐518. 24419115 10.1200/JCO.2013.51.7904 PMC5795691

[12] Bilimoria KY , Stewart AK , Winchester DP , Ko CY . The national cancer data base: a powerful initiative to improve cancer care in the United States. Ann Surg Oncol. 2008;15:683‐690. 18183467 10.1245/s10434-007-9747-3 PMC2234447

[13] van Gijn W , Marijnen CA , Nagtegaal ID , Kranenbarg EM , et al. Preoperative radiotherapy combined with total mesorectal excision for resectable rectal cancer: 12‐year follow‐up of the multicentre, randomised controlled TME trial. Lancet Oncol. 2011;12:575‐582. 21596621 10.1016/S1470-2045(11)70097-3

[14] Hall NR , Finan PJ , Al‐Jaberi T , Tsang CS , et al. Circumferential margin involvement after mesorectal excision of rectal cancer with curative intent. Predictor of survival but not local recurrence? Dis *Colon Rectum* . Dis Colon Rectum. 1998;41:979‐983. 9715152 10.1007/BF02237384

[15] Birbeck KF , Macklin CP , Tiffin NJ , Parsons W , et al. Rates of circumferential resection margin involvement vary between surgeons and predict outcomes in rectal cancer surgery. Ann Surg. 2002;235:449‐457. 11923599 10.1097/00000658-200204000-00001 PMC1422458

[16] Kapiteijn E , Marijnen CA , Nagtegaal ID , Putter H , et al. Preoperative radiotherapy combined with total mesorectal excision for resectable rectal cancer. N Engl J Med. 2001;345:638‐646. 11547717 10.1056/NEJMoa010580

[17] Habr‐Gama A , Perez RO , Kiss DR , Rawet V , et al. Preoperative chemoradiation therapy for low rectal cancer. Impact on downstaging and sphincter‐saving operations. Hepatogastroenterology. 2004;51:1703‐1707. 15532809

[18] Habr‐Gama A , Perez RO , Nadalin W , Sabbaga J , et al. Operative versus nonoperative treatment for stage 0 distal rectal cancer following chemoradiation therapy: long‐term results. Ann Surg. 2004;240:711‐717. 15383798 10.1097/01.sla.0000141194.27992.32 PMC1356472

[19] Dewdney A , Cunningham D , Tabernero J , Capdevila J , et al. Multicenter randomized phase II clinical trial comparing neoadjuvant oxaliplatin, capecitabine, and preoperative radiotherapy with or without cetuximab followed by total mesorectal excision in patients with high‐risk rectal cancer (EXPERT‐C). J Clin Oncol. 2012;30:1620‐1627. 22473163 10.1200/JCO.2011.39.6036

[20] Chua YJ , Barbachano Y , Cunningham D , Oates JR , et al. Neoadjuvant capecitabine and oxaliplatin before chemoradiotherapy and total mesorectal excision in MRI‐defined poor‐risk rectal cancer: a phase 2 trial. Lancet Oncol. 2010;11:241‐248. 20106720 10.1016/S1470-2045(09)70381-X

[21] Fernandez‐Martos C , Pericay C , Aparicio J , Salud A , et al. Phase II, randomized study of concomitant chemoradiotherapy followed by surgery and adjuvant capecitabine plus oxaliplatin (CAPOX) compared with induction CAPOX followed by concomitant chemoradiotherapy and surgery in magnetic resonance imaging‐defined, locally advanced rectal cancer: Grupo cancer de recto 3 study. J Clin Oncol. 2010;28:859‐865. 20065174 10.1200/JCO.2009.25.8541

[22] Andre T , Boni C , Navarro M , Tabernero J , et al. Improved overall survival with oxaliplatin, fluorouracil, and leucovorin as adjuvant treatment in stage II or III colon cancer in the MOSAIC trial. J Clin Oncol. 2009;27:3109‐3116. 19451431 10.1200/JCO.2008.20.6771

[23] Dossa F , Acuna SA , Rickles AS , Berho M , et al. Association between adjuvant chemotherapy and overall survival in patients with rectal cancer and pathological complete response after neoadjuvant chemotherapy and resection. JAMA Oncol. 2018;4:930‐937. 29710274 10.1001/jamaoncol.2017.5597 PMC6145724

[24] Polanco PM , Mokdad AA , Zhu H , Choti MA , et al. Association of adjuvant chemotherapy with overall survival in patients with rectal cancer and pathologic complete response following neoadjuvant chemotherapy and resection. JAMA Oncol. 2018;4:938‐943. 29710272 10.1001/jamaoncol.2018.0231 PMC6145733

[25] [dataset] https://www.facs.org/quality‐programs/cancer/ncdb/puf. Accessed May 22, 2018.

